# Evaluation of computed tomography post-processing images in postoperative assessment of Lisfranc injuries compared with plain radiographs

**DOI:** 10.1186/s13018-017-0589-9

**Published:** 2017-06-14

**Authors:** Haobo Li, Yanxi Chen, Minfei Qiang, Kun Zhang, Yuchen Jiang, Yijie Zhang, Xiaoyang Jia

**Affiliations:** Department of Orthopaedic Trauma, East Hospital, Tongji University School of Medicine, 150 Jimo Road, 200120 Shanghai, China

**Keywords:** Three-dimensional, Computed tomography, Computer-assisted, Lisfranc injury, Postoperative assessment

## Abstract

**Background:**

The objective of this study is to evaluate the value of computed tomography (CT) post-processing images in postoperative assessment of Lisfranc injuries compared with plain radiographs.

**Methods:**

A total of 79 cases with closed Lisfranc injuries that were treated with conventional open reduction and internal fixation from January 2010 to June 2016 were analyzed. Postoperative assessment was performed by two independent orthopedic surgeons with both plain radiographs and CT post-processing images. Inter- and intra-observer agreement were analyzed by kappa statistics while the differences between the two postoperative imaging assessments were assessed using the *χ* ^2^ test (McNemar’s test). Significance was assumed when *p* < 0.05.

**Results:**

Inter- and intra-observer agreement of CT post-processing images was much higher than that of plain radiographs. Non-anatomic reduction was more easily identified in patients with injuries of Myerson classifications A, B1, B2, and C1 using CT post-processing images with overall groups (*p* < 0.05), and poor internal fixation was also more easily detected in patients with injuries of Myerson classifications A, B1, B2, and C2 using CT post-processing images with overall groups (*p* < 0.05).

**Conclusions:**

CT post-processing images can be more reliable than plain radiographs in the postoperative assessment of reduction and implant placement for Lisfranc injuries.

## Background

Lisfranc injury, also known as tarsometatarsal joint fracture-dislocation, accounts for approximately 1% of all orthopedic trauma [1]. As the connection between the forefoot and midfoot, Lisfranc joints play a significant role on stress transduction and foot stability. Once a Lisfranc injury occurs, malunion and traumatic arthritis can develop if treatments are not properly performed [2, 3]. Early stable anatomical reduction for Lisfranc injuries is necessary [4, 5] and precise postoperative assessment guarantees an opportunity to perform early intervention and follow-up before adverse functional outcomes develop.

As major approaches of diagnosing and postoperative assessment for Lisfranc injuries, plain radiographs in dorsal anteroposterior and oblique position of foot and CT scanning of whole foot are important. For Lisfranc injuries, traditional plain radiographs cannot offer precise images due to interference created by an incorrect projection angle of the tube, ankle swelling, or image overlap. While more details of fractures can be shown using CT images for precise diagnoses, the application of original CT images during postoperative assessment is still not sufficient due to the interference of metal artefacts that are generated by implants.

CT post-processing imaging techniques, a series of multiple imaging techniques using original CT data for 2D or 3D reconstruction of bones, have been increasingly applied. At present, the CT post-processing technique was sufficiently accepted and used in preoperative planning of Lisfranc injuries [6–10]; however, the application of CT to the postoperative assessment of Lisfranc injuries is not yet fully developed. In this study, the postoperative assessment value of CT post-processing images was evaluated using multiple imaging techniques, including shaded surface display (SSD), volume-rendering (VR), and multiplanar reconstruction (MPR), and was compared with plain radiographs.

## Methods

### Patients

From January 2010 to June 2016, postoperative CT original data of 79 cases (43 males and 36 females) were collected. All patients were aged from 25 to 64 years (35.6 years old in average). We excluded deformity of the feet, osteoarthritis, or bone tumors. The groups were divided according to the Myerson classification [11], with 17 cases in type A, 23 cases in type B1, 26 cases in type B2, 8 cases in type C1, and 5 cases in type C2. Patients with CT records from more than one assessment were included only once. All cases were closed injuries that were treated by operation reduction and internal fixation within 2 weeks. Prior to radiography and CT scanning, informed consent was obtained from all participants, and the study was approved by the institutional review board of the hospital, which conforms to the provisions of the Declaration of Helsinki.

### Surgical treatment

After intraspinal or general anesthesia, operations were performed with incisions made according to Myerson classification. Patients with an injury of Myerson classification A, a dorsal longitudinal incision centered over the second and fourth metatarsals and an incision between the fourth and fifth metatarsals were selected, which allowed for adequate exposure of the medial and middle columns. The second tarsometatarsal joint was reduced first. Generally, once the base of the second metatarsal had been reduced anatomically, the first metatarsal was positioned correctly on the medial cuneiform [12, 13]. The remaining tarsometatarsal joints were then easily reduced. The base of the third metatarsal was fixed to the intermediate or lateral cuneiform while the base of the fourth and the fifth metatarsals were fixed to the lateral cuneiform. For patients with an injury of Myerson classification B1, a dorsal incision of the first tarsometatarsal joint was performed, with a dorsal plate often being used for reduction. For patients with Myerson classification B2, we selected an incision between the second and third metatarsals. As for patients with Myerson classification C1 or C2, the approach for reduction was mostly the same with that for patients of type A [14]. The choosing of internal fixation depended on the stability it could contribute to the reduction individually. In some cases, when fractures of other parts such as the distal part of metatarsals also existed, reduction and internal fixation were also done.

### Image post-processing and assessment

All data were collected from the department of radiology of the hospital, which were saved in the DICOM 3.0 format (.dcm). CT was performed using a 16–detector row CT scanner (GE Light-Speed CT; Waukesha, WI, USA). Imaging parameters for CT scanning were as follows: section thickness, 0.625 mm; tube voltage, 120 kVp; pitch, 1.375; matrix, 512 × 512. Thin-slice CT transverse images of all subjects were first uploaded to the PACS, with the CT data (DICOM 3.0) then being inputted into a computer-aided, orthopedic clinical research platform (SuperImage orthopedics edition 1.0, Cybermed Ltd, Shanghai, China) via removable storage devices. The bone and non-bone materials were defined by assigning a CT density threshold (of Hounsfield units). The 3D structures of each bone consisted of Lisfranc joints and were reconstructed using a shaded surface display (SSD) with a reconstruction interval of 0.625 mm and a density threshold of 150H. A 3D interactive and automatic segmentation technique was applied to distinguish all component bones [15–18]. According to the literatures about the criteria of the reduction for Lisfranc injuries [7, 8, 11], postoperative evaluation of the reduction was performed using the below-described criteria. Angulation of the metatarsals with the talus beyond 15°, diastasis that was greater than 2 mm between the base of the first and second metatarsals, or fracture fragment displacement beyond 2 mm, were considered not acceptable as forms of stable anatomical reduction. Regarding implant placement, unsuitable placements of the implant or screw with 30% of its length exposed into joint cavity or through the joint surface were not acceptable [17]. The assessment was performed on two separate occasions at an interval of 4 weeks by two independent orthopaedic surgeons with image-reading experience of 12 and 3 years. The plain radiographs and CT post-processing images were assessed separately. If there was disagreement, a third orthopedist with 15 years of experience in image reading was consulted (Table 1).

**Table 1:** The criteria for non-anatomical reduction and poor internal fixation

Criteria	Non-anatomical	Poor internal fixation
1	Diastasis between the base of the first and second metatarsals >2 mm	Placement of implantation is inappropriate
2	Angulation of the first metatarsal with the talus > 15°	Exposed part of the screw is >30%
3	Fracture fragment displacement is >1 mm	Screw penetrates into articular cavity or through articular surface

### Statistical analysis

Statistical analysis was performed using SPSS 22.0 (SPSS, Chicago, IL, USA). Kappa statistics were applied to inter- and intra-observer variations with values between 1.00 and 0.81 indicating perfect accord, between 0.80 and 0.61 indicating substantial accord, between 0.60 and 0.41 indicating moderate accord, between 0.40 and 0.21 indicating fair accord, between 0.20 and 0.00 indicating slight accord, and below 0.00 indicating poor accord [14]. The difference between plain radiographs and CT post-processing images in the postoperative assessment was evaluated using a *χ* ^2^ test (McNemar’s test), with the assumption that *p* < 0.05 was indicative of significant.

## Results

Kappa statistic is the most commonly used statistic for the agreement between two observers that takes into account the fact that observers will sometimes agree or disagree simply by chance. According to the kappa statistics, inter- and intra-observer agreement of CT post-processing images (0.866–0.969) was much higher than that of plain radiographs (0.473–0.786) in the evaluation of the quality of anatomical reduction (Table 2). In the aspect of internal fixation quality, significant differences were also observed in inter- and intra-observer agreement between CT post-processing images (0.843–0.935) and plain radiographs (0.487–0.794) (Table 2).

**Table 2:** Inter- and intra-observer agreement of CT post-processing images and plain radiographs in kappa values

	Plain radiographs				CT post-processing images			
	Inter-observer		Intra-observer		Inter-observer		Intra-observer	
	First reading	Second reading	Examiner 1	Examiner 2	First reading	Second reading	Examiner 1	Examiner 2
Diastasis between the base of the first and second metatarsals	0.547	0.473	0.647	0.786	0.969	0.935	0.968	0.898
Angulation of the metatarsals with the talus	0.473	0.647	0.737	0.552	0.907	0.909	0.874	0.901
Fracture fragment displacement	0.687	0.640	0.647	0.749	0.877	0.907	0.866	0.901
Screw exposure	0.487	0.661	0.661	0.490	0.856	0.890	0.843	0.928
Screw penetration	0.661	0.661	0.794	0.661	0.904	0.866	0.933	0.935

**Fig. 1:**
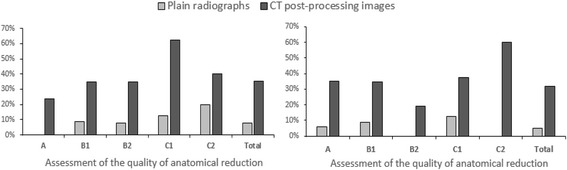
The comparison between plain radiographs and CT post-processing images in the assessment of postoperative Lisfranc injuries (percentage of the non-anatomical reduction and poor internal fixation)

In the assessment of the quality of anatomical reduction, 0/17 (0.00%), 2/23 (8.70%), 2/26 (7.69%), 1/8 (12.50%), and 1/5 (20.00%) cases with Myerson classifications of A, B1, B2, C1, and C2, respectively, were identified as having undergone a poor reduction. Meanwhile, CT post-processing images indicated that 4/17 (23.53%), 8/23 (34.78%), 9/26 (34.62%), 5/8 (62.50%), and 2/5 (40.00%) cases with Myerson classifications of A, B1, B2, C1, and C2, respectively, were identified as having undergone a poor reduction (Fig. 1). Significant differences between CT post-processing images and plain radiographs were observed in Myerson classifications of A, B1, B2, and C1 (*p* < 0.05), while the overall difference was significant (*χ* ^2^ = 16.19; *p* < 0.05). All poor reduction cases that were observed using plain radiographs were also identifiable by CT post-processing images; however, the converse was found to not be true.

Regarding internal fixation qualities, plain radiographs revealed poor internal fixation in 1/17 (5.88%), 2/23 (8.70%), 0/26 (0.00%), 1/8 (12.50%), and 0/5 (0.00%) of cases with Myerson classifications of A, B1, B2, C1, and C2, respectively. Meanwhile CT post-processing images identified poor internal fixation in 6/17 (35.29%), 8/23 (34.78%), 5/26 (19.23%), 3/8 (37.50%), and 3/5 (60%) cases with Myerson classifications of A, B1, B2, C1, and C2, respectively. Significant differences between CT post-processing images and plain radiographs were observed in Myerson classifications of A, B1, B2, and C2 (*p* < 0.05), as well as in the overall evaluation (*χ* ^2^ = 18.63, *p* < 0.05) (Fig. 1). All poor internal fixation cases that were observed using plain radiographs were also identifiable by CT post-processing images; however, the converse was found to not be true. CT post-processing images identified more cases with poor internal fixation than plain radiographs did.

## Discussion

The Lisfranc joint connects the forefoot and midfoot. Accordingly, any kind of injuries may seriously affect the configuration and mechanical transduction of feet. Therefore, early anatomic reduction is required for the recovery of stress transduction in the foot and walking function [1–3, 5]. Anatomical reduction and stable implant placement are based on precise preoperative planning and postoperative evaluation. Radiographs represent a key tool for such evaluations. As the Lisfranc joint consists of several bones with irregular shapes and narrow joint space, traditional plain radiographs cannot offer precise images due to interference created by an incorrect projection angle of the tube, ankle swelling, or image overlap. So far, CT images, offering more details of fractures, has been widely used in improving the precision of diagnoses and preoperative planning [6–10]. However, the application of original CT images on postoperative assessment is still not generalizable because of the interference of metal artefacts that are generated by implants on axial CT images.

With the imaging techniques developing, CT post-processing images, using original CT data for 2D or 3D reconstruction of bones, can make full use of the CT for diagnosing, preoperative planning, and postoperative assessment. In our study, multiple imaging techniques based on CT original data were applied for postoperative assessment of Lisfranc injuries. In using SSD, MPR and VR [19, 20], each of which has unique advantages, we found that multiple imaging methods offered more approaches for postoperative assessment [21, 22]. SSD was the first 3D rendering technique applied to medical imaging and was mainly applied in orthopaedics because of its superiority for bony surface reconstructions. It presents a 3D reconstruction of the surface of bone based on pixel thresholds of the CT original data, which could offer stereoscopic images of components of the bones and non-anatomic reduction bone fragments of the Lisfranc joint [12]. MPR is a multi-planar 2D reconstruction that involves processed axial CT images. A volume is built by stacking the axial slices. The software then cuts slices through the volume in a different plane. In the MPR mode, additional details of the internal structure of bones and the joint space could be figure out [23, 24]. The VR technique images tissue by measuring the transparency of different tissues. In volume rendering, transparency, colors and shading are used to allow a better representation of the volume to be shown in a single image, thus presenting clearer spatial relationship of different tissues. Moreover, the assessment for implant placement can also be determined using its enhanced imaging and eliminating metal artefacts for implant. By combining VR and MPR, non-anatomic reduction can be performed more precisely. In comparison, traditional radiography based on X-ray imaging is usually unable to obtain accurate information of complete fracture displacement and of the injury at the joint surface. The advantage of multiple imaging techniques based on CT datasets is not its ability to define new observations, but rather its reproducibility and clarity.

**Fig. 2:**
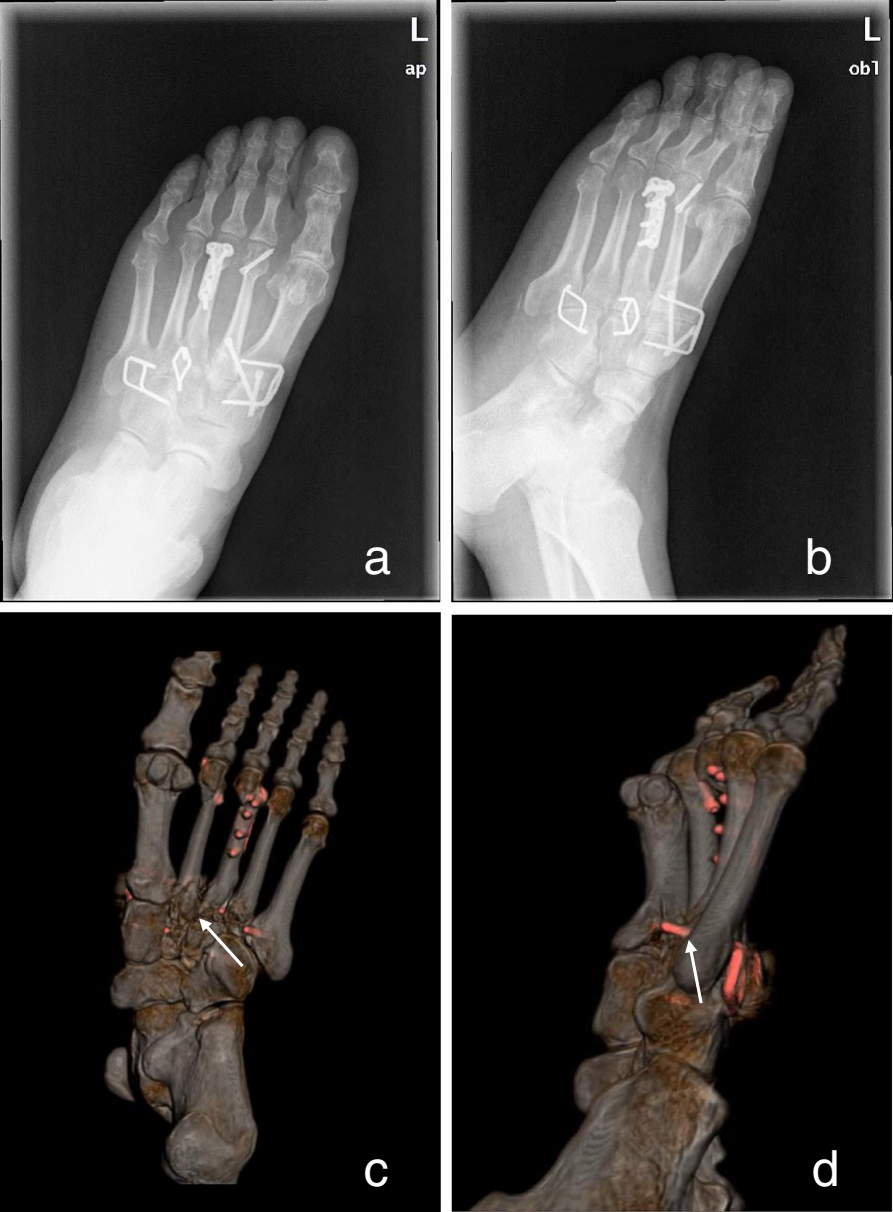
A 50-year-old man with Lisfranc injury of type C2. The anteroposterior and oblique plain radiograph (**a**, **b**) showed an anatomical reduction and fine international fixation. But in the VR model of CT images (**c**, **d**), a fracture fragment was found poor reduction with a distance of more than 1 mm in image **c**, while from the lateral view (**d**), the screw placed into the fifth metatarsal base was found with an exposed part of more than 30% of the screw

**Fig. 3:**
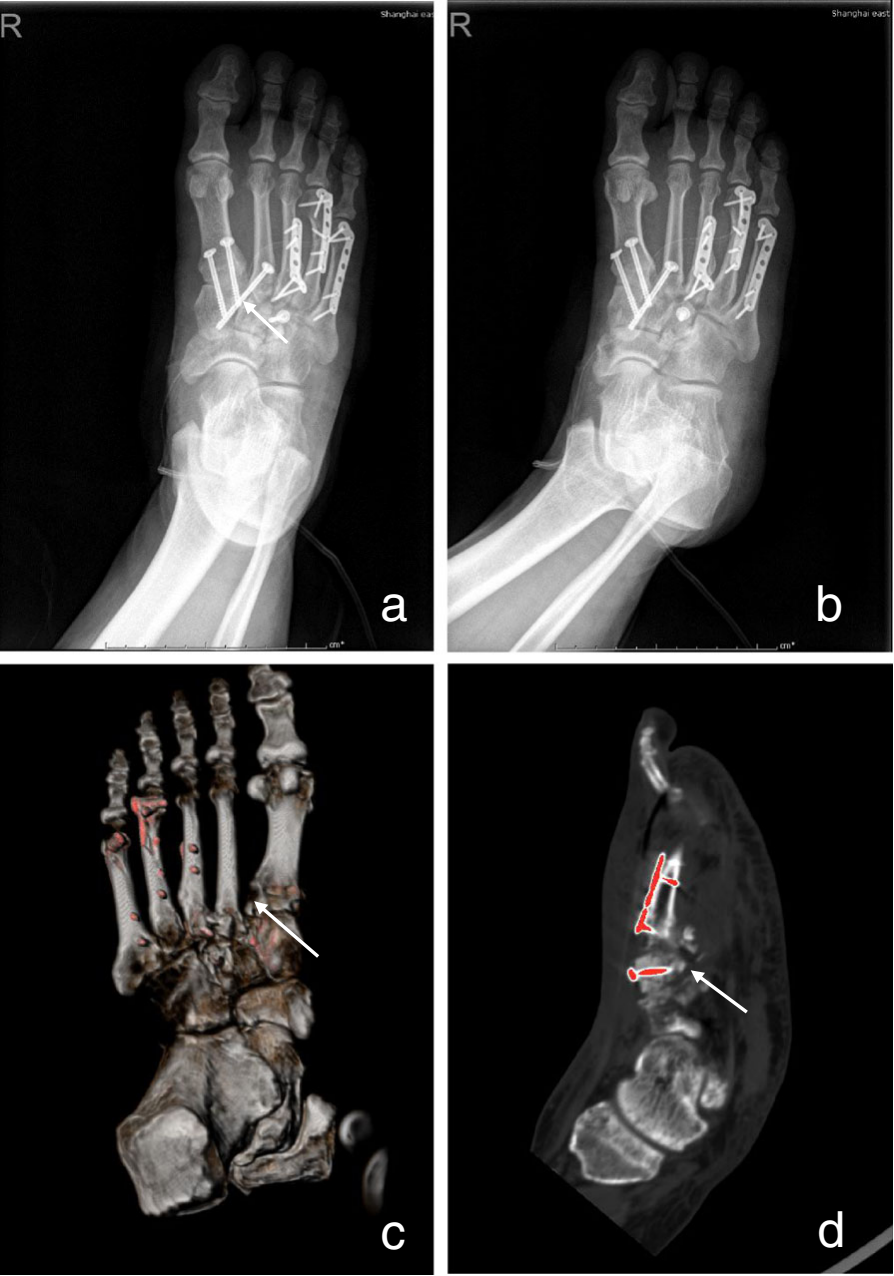
A 53-year-old man with a Lisfranc injury of Myerson classification C1. The postoperative anteroposterior and oblique plain radiograph (**a**, **b**) showed the reduction was poor because of the diastasis between the base of the first and second metatarsals was beyond 2 mm, which was also showed clearly in the post-processing CT images in VR mode (**d**). But furthermore, in the MPR mode of CT images, more fracture fragments at the second and third metatarsal bases were found not reducted with a distance of more than 1 mm (**c**), while could not be discovered clearly in the plain radiographs

Regarding the assessment of reduction in this study, more non-anatomic reductions were determined in injuries of types A, B1, B2, and C1, whereas more bone fragments and steps of articular surface, particularly at the second metatarsal base, were found using CT post-processing images (Figs. 2 and 3). Compared with plain radiographs, CT post-processing images processed using SSD, VR, and MPR showed more details of the internal structure and articular surface, thereby indicating its precise postoperative assessment. According to the above results, we found that non-anatomical reduction that is detectable using plain radiographs can also be detected using CT post-processing images; however, the converse was not always true. Using CT post-processing images has a more valuable role in evaluating the quality of the reduction of Lisfranc injuries. Identifying patients with non-anatomical reduction as soon as possible can be significant. With non-anatomical reduction being figured out at early stage, subsequent clinical management can be adjusted, such as carrying out more frequent follow-up examinations. Thus, early intervention to prevent osteoarthritis could be achieved, which can prevent cases of malunion.

**Fig. 4:**
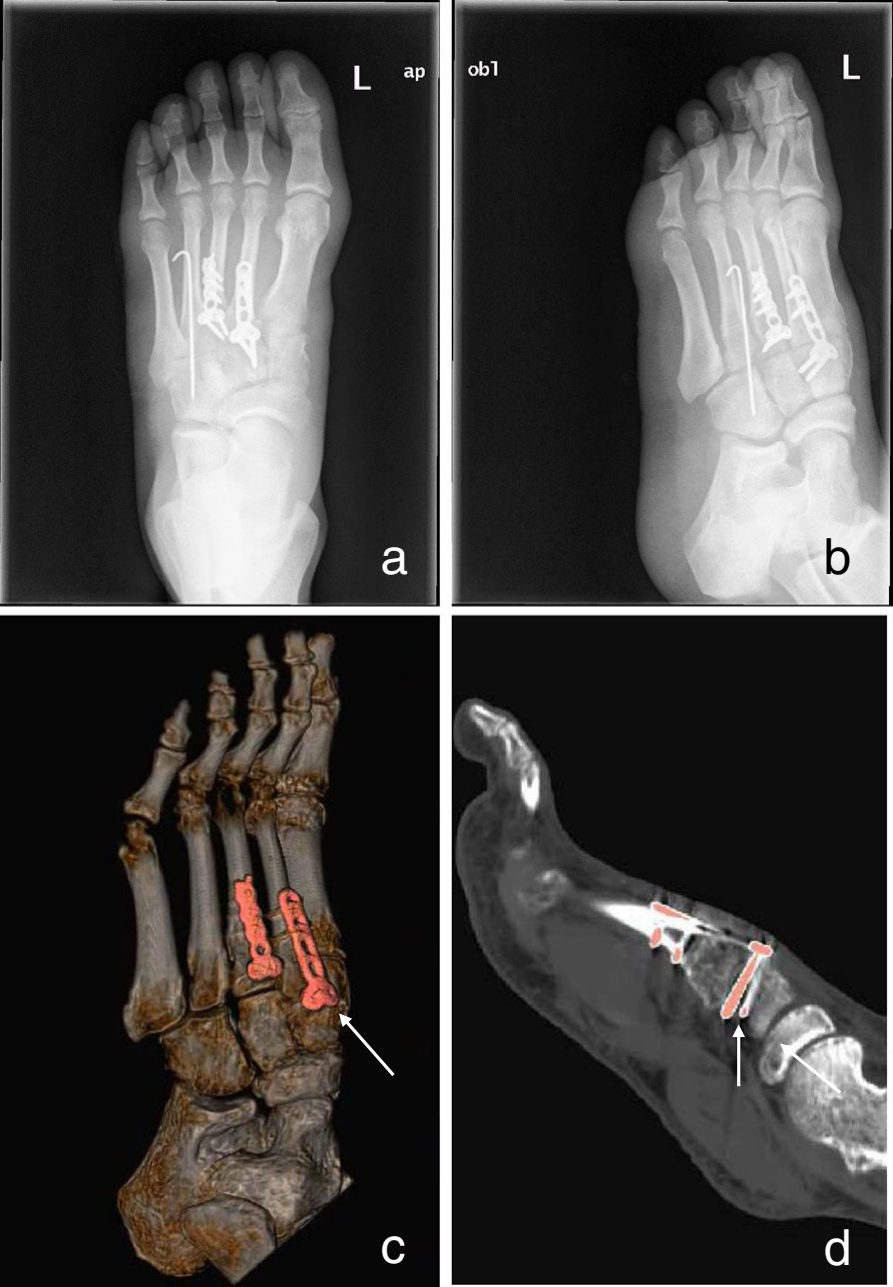
A 36-year-old woman with a Lisfranc injury of Myerson classification B2. In the anteroposterior and oblique plain radiograph (**a**, **b**), the internal fixation was placed well. But in the VR mode of CT images, the position of the screws at the second metatarsal base was found like placed into the articular cavity (**c**), and we checked the image of the articular surface in the MPR mode further and found that one of the screws was place into the articular cavity definitely (**d﻿**)

As for the assessment of internal fixation, we observed significant differences in detecting implant placement between CT post-processing images and plain radiographs in patients with injuries of Myerson classifications A, B1, B2, and C2. Similar differences were found in the evaluation of the quality of internal fixation. Namely, CT post-processing images was better able to detect obscure inappropriate internal fixation. Combined VR and MPR, particularly after eliminating metal artefacts, allowed for enhanced imaging of implants and subsequently better detection of inappropriate international fixation, particularly over the length of the screws and those that were placed into the articular cavity (Figs. 2 and 4). Precise detecting of poor internal fixation can help with the guide of hardware removal. Although improvement in pain relief and function can be expected after hardware removal from the pain region of the fracture fixation, the clinical indications for implant removal are not well established [12]. Some factors such as the cost of the procedure, work time lost, and other potential causes of pain, such as infection and non-union, may also influence the clinical decision. Once poor internal fixation could be observed clearly on CT post-processing images, it would be easier to make an informed clinical decision.

There are some limitations to this study. The cross-sections in this study were redefined, which were presented for the first time. The study is the first to provide detailed data of the assessment of the operational outcomes of Lisfranc injuries based on multiple imaging measurements. However, the software we applied is not widely used. Therefore, the veracity and rationality of such imaging processing requires further study and continuous improvements. Second, this study is not a multicenter study. Therefore, the results can only show the ability of our own study group.

## Conclusions

In conclusion, we found greater value in CT post-processing images in evaluating the operational outcomes Lisfranc fracture treatment. Compared with plain radiographs, more details are visible using CT post-processing images both in the evaluation of anatomical reduction and of internal fixation. Furthermore, CT post-processing images were also more reliable than plain radiographs according to inter- and intra-observer agreement. With a better postoperative assessment of Lisfranc fractures using CT post-processing images, early intervention and follow-ups can be performed. In doing so, clinicians can address non-anatomical reductions and poor internal fixations, thus performing early removal of implants in cases of poor internal fixation, which can prevent osteoarthritis. Therefore, based on our findings, it is recommended that the CT post-processing imaging method be used during postoperative evaluation of Lisfranc fracture treatment.

## Abbreviations

3D: Three-dimensional
CT: Computed tomography
DICOM: Digital Imaging and Communication in Medicine
MPR: Multiplanar reconstruction
SSD: Shaded surface display
VR: Volume-rendering
